# Impacts of climate change on climatically suitable regions of two invasive *Erigeron* weeds in China

**DOI:** 10.3389/fpls.2023.1238656

**Published:** 2023-09-28

**Authors:** Yumeng Huang, Guoliang Zhang, Weidong Fu, Yue Zhang, Zihua Zhao, Zhihong Li, Yujia Qin

**Affiliations:** ^1^ Key Laboratory of Surveillance and Management for Plant Quarantine Pests, Ministry of Agriculture and Rural Affairs, College of Plant Protection, China Agricultural University, Beijing, China; ^2^ Institute of Environment and Sustainable Development in Agriculture, Chinese Academy of Agricultural Sciences, Beijing, China

**Keywords:** *Erigeron philadelphicus*, *Erigeron annuus*, maxent, potential geographical distribution, distribution pattern

## Abstract

**Introduction:**

*Erigeron philadelphicus* and *Erigeron annuus* are two ecologically destructive invasive plants from the Asteraceae family. Predicting the potential distribution pattern of two invasive alien *Erigeron* weeds can provide a scientific basis for prevent the further spread of these two weeds in China under climate change.

**Methods:**

Based on historical occurrence datasets and environmental variables, we optimized a MaxEnt model to predict the potential suitable habitats of *E. philadelphicus* and *E. annuus*. We also analyzed the shifts of distribution centroids and patterns under climate change scenarios.

**Results:**

The key variables that affect the potential geographical distribution of *E. annuus* and *E. philadelphicus*, respectively, are temperature seasonality and precipitation of the driest month. Moreover, topsoil sodicity and topsoil salinity also influence the distribution of *E. philadelphicus*. Under climate change, the overall suitable habitats for both invasive alien *Erigeron* weeds are expected to expand. The potential geographical distribution of *E. annuus* exhibited the highest expansion under the SSP245 climate scenario (medium forcing scenarios), whereas *E. philadelphicus* had the highest expansion under the SSP126 climate scenario (lower forcing scenarios) globally. The future centroid of *E. annuus* is projected to shift to higher latitudes specifically from Hubei to Hebei, whereas *E. philadelphicus* remains concentrated primarily in Hubei Province. The overlapping suitable areas of the two invasive alien *Erigeron* plants mainly occur in Jiangsu, Zhejiang, Fujian, Jiangxi, Hunan, Guizhou, and Chongqing, within China.

**Discussion:**

Climate change will enable *E. annuus* to expand into northeastern region and invade Yunnan Province whereas *E. philadelphicus* was historically the only suitable species. *E. annuus* demonstrates a greater potential for invasion and expansion under climate change, as it exhibits higher environmental tolerance. The predictive results obtained in this study can serve as a valuable reference for early warning systems and management strategies aimed at controlling the spread of these two invasive plants.

## Introduction

The scientific review published by the IPCC in 2021 highlights the significant impact of global climate change on the spread of pests (IPCC, 2021). The global spread of pests is driven by both anthropogenic factors, such as global travel and trade and natural factors. Among the natural factors, global climate change plays a crucial role (Turbelin et al., 2017; Trisos et al., 2020; Clavijo et al., 2023). The global spread of pests has led to an increase in biological invasions. Biological invasions have become a severe issue worldwide, and climate warming has been identified as a factor influencing these phenomena (Cornelissen et al., 2019). The impact of invasive alien plant species on food chains is increasingly being observed in many countries (Becker et al., 2005; Clavijo et al., 2023). In China, more than 660 invasive alien species have been reported, with invasive alien plants accounting for 45% of them (Wan et al., 2017). The Asteraceae family boasts the highest number of invasive alien plants in China, thanks to characteristics such as a length flowering period, non-fusion reproduction or self-fertilization, sufficient pollinators, high seed production, and high seed germination rates, with each of these characteristics inclined to increase invasiveness (Chen et al., 2018).

The genus *Erigeron* consists of over 200 species in total, primarily distributed in Europe, mainland Asia, and North America, with a few species found in Africa and Oceania. China is home to 35 species, mainly concentrated in Xinjiang and the southwestern mountainous regions (Editorial Committee of Flora of China of Chinese Academy of Sciences, 1985). *E. annuus* (annual fleabane) is recognized as one of the most serious invasive alien plants in China, with a wide distribution and a highly competitive nature (Zhang, 2011a). The Ministry of Ecology and Environment of China listed *E. annuus* as an invasive alien species (Fan et al., 2020). Notably, *E. annuus* displays remarkable adaptability to many environments, capable of flourishing even on stone walls. It exhibits a strong reproductive ability with a large seed yield and is easily spread by the wind due to its characteristic hairy seeds (Huang, 2011). Originating from North America (Sennikov and Kurtto, 2019), *E. annuus* was initially discovered in Shanghai, China, in the late 19th century. Presently, it is encountered in Hebei, Henan, Jiangsu, Zhejiang, Shandong, Anhui, Fujian, Jiangxi, and Hubei, with worldwide distribution concentrated in the northern hemisphere, i.e., North America, Europe, and Asia, and particularly concentrated in temperate and subtropical coastal areas (Zhang and Wang, 2009). It has also been observed extensively in urban areas and poses a significant threat to vegetable patches including maize fields as well as cash crop areas such as orchards, tea gardens, mulberry gardens, and nurseries (Rutkowski, 2004; Li, 2020). It alters the environment by releasing chemosensitive substances, resulting in the suppression of the growth of native plants (Li et al., 2007; Zhang, 2008). Furthermore, its rapid growth rate, large number of seeds, and ease of dissemination allow it to spread extensively, threatening native plant survival and reducing biodiversity and ecosystem stability (Huang, 2011).

*E. philadelphicus*, also known as Philadelphia philadelphus or spring philadelphus, is another invasive alien plant within the genus *Erigeron* (Wang et al., 2016). It is morphologically diverse, and suitable for low-temperature, mesic or humid environments, and is resistant to drought, but it is not saline-tolerant (Wang, 2008). Similarly originating from North America, it was first found in China in the lower reaches of the Yangtze River and is now widely distributed in Jiangsu, Zhejiang, Shanghai, and other nearby regions. It is primarily distributed in North America, Europe, and the Eurasian continents on a global scale (Wang, 2008). It is similarly destructive to *E. annuus*, which can form dense population patches of 300–400 m^2^ (Zhang et al., 2011b). Improved monitoring and prevention efforts are essential to mitigate further losses.

Predicting the potential geographical distribution is a crucial approach of pest risk analysis that aims to predict the suitable range and degree of pests in the study area by arranging distribution information, biological information, and climate geographic data (Li, 2015; McMahon et al., 2021). The maximum entropy model (MaxEnt) is widely used in predicting species distribution based on known information about species distributions and climate variables (Jaynese, 1957). It was initially proposed by Phillips and Dud´ık (Dud´ık et al., 2004; Phillips et al., 2006), and is becoming popular and widely used in the field of predicting species distribution. Compared with other models, the MaxEnt model has some advantages, such as supporting a small sample size and having high complexity and better model performance (Xiong et al., 2004; Kumar and Stohlgren, 2009). The latitude and longitude of the population distribution centers can be obtained using ArcGIS tools, which aids in predicting population dispersal trends. A combination of these two methods has been widely used, such as for predicting species distribution for *Codonopsis pilosula* (Yan et al., 2021), *Lolium temulentum* L. (Yang et al., 2022), *Solidago canadensis* (Phillips et al., 2006)*, Ageratina adenophora* (Poudel et al., 2020), and *Mikania micrantha* (Rameshprabu & Swamy, 2015).

The current study utilized the known distribution records and related climatic variables, combined with MaxEnt and ArcGIS, to predict the shifts of potential suitable habitats, distribution centroids, and the distribution pattern of two invasive alien *Erigeron* weeds (*E. philadelphicus* and *E. annuus*) in China, aiming to provide a scientific basis for effective early warning management in China under climate change.

## Materials and methods

### Distribution records of Erigeron philadelphicus and Erigeron annuus

The distribution records of *E. annuus* and *E. philadelphicus* were accessed and downloaded from the Center for Agriculture and Bioscience International (CABI, 2022a; CABI, 2022b), the Global Biodiversity Information Facility (GBIF, 2022a; GBIF, 2022b), the China Digital Herbarium (https://www.cvh.ac.cn/, accessed April 2023), and related literature (Zhang et al., 2011b; Fan et al., 2020; Sennikov et al., 2020). We imported all the distribution points collected into ArcGIS, referred to relevant literature, and removed unreasonable data and distribution points with missing environmental data. Spatial filtering was applied using the spThin function in RStudio 4.1.3 to minimize spatial autocorrelation. Following this filtering, the remaining distribution points were separated by a minimum distance of 9 km, indicating their suitability for model building. After filtering the raw data, a total of 2,279 valid distribution records for *E. philadelphicus* and 793 distribution points for *E. annuus* were retained.

### Environmental variables

We obtained elevation (ele) data and historical (1970–2000) and future (2040–2060) (the 2050s) data for 19 bioclimatic variables (bio1–bio19) in Worldclim version 2.1 (https://www.worldclim.org, accessed May 2023), with a spatial resolution of 5 arc_min. For future environmental data, we selected four different Shared Socio-economic Pathways in 2050 under the BCC-CSM2-MR model: SSP126, SSP245, SSP370, and SSP585. SSP126 means lower forcing scenarios (radiative forcing reaches 2.6 W/m^2^ in 2100; the temperature may be lower than 2.0°C relative to the pre-industrial revolution). SSP245 and SSP370 mean medium forcing scenarios (radiative forcing becomes 4.5 and 7.0 W/m^2^ in 2100). SSP585 means the highest forcing scenarios (radiative forcing reaches 8.5 W/m^2^ in 2100) (Lan et al., 2022). Soil salinity data (Esp and Ece) were provided by the FAO World Soil Database version 1.2 (http://www.fao.org/soils-portal/soil-survey/soil-maps-and-databases/har-monized-world-soil-database-v12/en/, accessed May 2023). The collinearity of environmental variables may influence parameter optimization during the calibration process of the MaxEnt model (Dormann et al., 2013). The variable screening process was as follows: (1) we imported 22 environmental variables and the current distribution into MaxEnt to remove zero-contribution rate variables; (2) the sampling function of ArcGIS was used to extract the environmental data of all distribution points; and (3) to reduce the collinearity between highly correlated variables, correlation analysis and principal component analysis (PCA) were processed by SPSS version 25.

### MaxEnt model optimization

To optimize the model and prevent overfitting, it is important to set relevant parameters (Zhao et al., 2022). The regularization multiplier (RM) and feature combinations (FCs) are regarded as two critical parameters for optimal settings in model calibration (Muscarella et al., 2014), which can be selected in the basic settings. The feature combinations represent different transformations of the covariable (Lu et al., 2020) including linear features (L), quadratic features (Q), product features (P), hinge features (H), and threshold features (T) (Muscarella et al., 2014). RM was set from 0.5 to 4, with a 0.5 interval, and six different FC combinations, namely, L, LQ, LQP, LQH, LQHP, and LQHPT. We utilized the R package (ENMevals) to select the FC and RM values as the optimization parameters for subsequent prediction when the optimized minimum information criterion (Delta AICc) is 0 (Warren & Seifert, 2011; Ye et al., 2021). The results showed that RM was 0.5 for both *E. philadelphicus* and *E. annuus*, and the FCs were LQPTH.

### Model settings and assessment

The distribution records and environmental variables obtained for the two species were separately imported into MaxEnt 3.4.4. In the modeling, 25% of the data were used to test the model and 10 repetitions were set for each type of subsample. The maximum number of iterations was 5,000, and the 10 percentile training presence was added. We selected environmental parameters using Jackknife analysis. This analysis is commonly used to analyze the influence of various environmental variables on the predictive outcomes or to determine the key environmental factors affecting species distribution (Peterson & Cohoon, 1999; Phillips et al., 2004). By using the Jackknife analysis, the primary environmental variables that affect the distribution of a species can be analyzed. The principle behind this method is to sequentially omit each environmental variable during the computation, build a model using the remaining variables, and then analyze the correlation between the omitted environmental variable and the omission error. If the absence of an environmental variable leads to a significant increase in omission error, it indicates that this environmental parameter significantly affects the predictive outcomes of the model (Peterson et al., 2007). The RM was set to 0.5, and the FC was set to LQPTH. The results from MaxEnt were imported into ArcGIS and converted to Raster. The map displaying suitable habitats was classified into four categories: negligible risk, low risk, medium risk, and high risk. Jenks Natural Break Classification (NBC) was used in this study, and this method minimizes the variation within each range, so the areas are as close as possible in value to each other (Gao & Shi, 2021; Zhang et al., 2022b). According to the result plots from MaxEnt, the classification for *E. philadelphicus* was 0–0.12, 0.12–0.31, 0.31–0.69, and 0.69–1, and that for *E. annuus* was 0–0.08, 0.08–0.31, 0.31–0.69, and 0.69–1 with *Fixed cumulative value 5 Cloglog threshold*. In this study, the area under the receiver operating characteristic (ROC) curve, also known as the area under the curve (AUC), was chosen as the evaluation metric. The AUC value ranges from 0 to 1, which can be interpreted as follows: 0 to 0.5 (prediction no better than random); 0.5 to 0.7 (average prediction effect); 0.7 to 0.9 (good prediction effect); and 0.9 to 1.0 (excellent prediction effect) (Franklin & Miller, 2010). The data from different climatic conditions were reclassified and assigned to obtain future changes in the suitable habitats for the four climatic scenarios compared to historical conditions using the raster calculator. The raster to point tool was used and the mean distribution centroid was calculated for each climate condition.

## Results

### Shifts in potential suitable habitats

**Figure 1:**
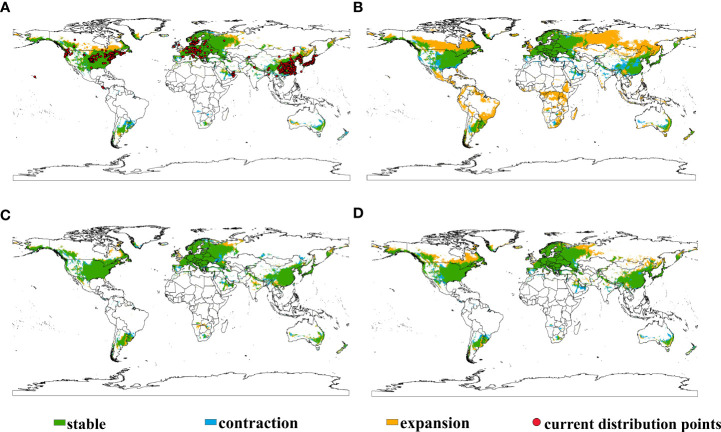
Shifts in potential suitable habitats of *Erigeron annuus* under different climate scenarios globally. **(A)** Current to SSP126, **(B)** current to SSP245, **(C)** current to SSP370, and **(D)** current to SSP585.

The study examined the shifts of the global potential suitable habitats of *E. annuus* under historical climate condition and different future climate scenarios in 2050 (**Figure 1**; **Table 1**). Results revealed that the potential suitable habitats of *E. annuus* were mainly concentrated in central and coastal North America, Europe, eastern Asia, southeastern parts of South America, southern Australia, and southern Africa (**Figures S2, S3**). The analysis further predicted the expansion of potential suitable habitats under all future scenarios, with the largest expansion under the SSP245 climate scenario, mainly in Asia, Africa, and America. Under future climate conditions, the native range in North America shows an expansion trend, especially under the SSP245 climate scenario, where there is a large-scale expansion of suitable habitats. In this climate scenario, apart from North America, the introduced ranges in Europe and Asia also experience significant expansion. Additionally, Africa and South America, where the species is currently not distributed, show notable expansion under the SSP245 climate scenario.

**Table 1:** Dynamics of the potential suitable habitats area of *Erigeron annuus* and *Erigeron philadelphicus* under different climate scenarios globally (compared to historical climate conditions).

Species	Climate scenario	Area (×10^4^ km^2^)			
		Expansion	Contraction	Stable	Change
*Erigeron annuus*	2050-SSP126	692.70	409.58	2,880.59	283.12
	2050-SSP245	3105.75	617.75	2,672.52	2488
	2050-SSP370	491.86	452.38	2,837.80	39.48
	2050-SSP585	969.92	432.58	2,857.59	537.34
*Erigeron philadelphicus*	2050-SSP126	522.26	175.20	3279.17	347.06
	2050-SSP245	451.36	222.41	3231.96	228.95
	2050-SSP370	469.65	250.01	3204.37	246.64
	2050-SSP585	502.91	303.96	3150.42	198.95

**Figure 2:**
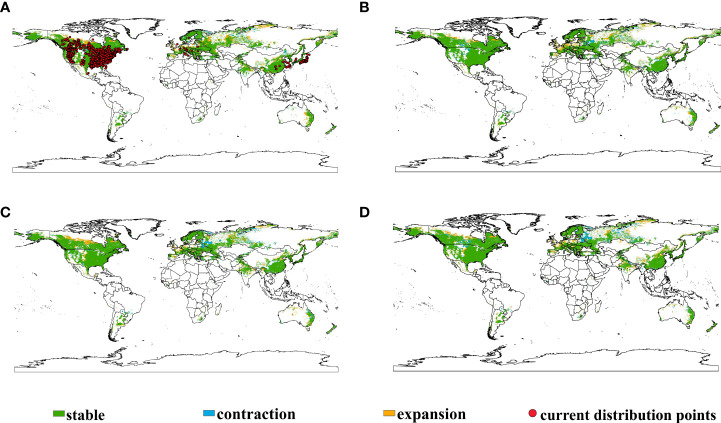
Shifts in potential suitable habitats of *Erigeron philadelphicus* under different climate scenarios globally. **(A)** Current to SSP126, **(B)** current to SSP245, **(C)** current to SSP370, and **(D)** current to SSP585.

The analysis of potential suitable habitat shifts under historical condition and different future climate scenarios in 2050 (**Figure 2**; **Table 1**) revealed that the main distribution of *E. philadelphicus* was predominantly inhabited in subtropical areas at 30°–40° N latitude: encompassing native North America, eastern Asia, Europe, eastern Australia, small parts of South America, and Africa (**Figure S6**). Although an overall expansion trend was noted for *E. philadelphicus*, the increase in suitable habitats was not apparent across the four future climate scenarios (**Figure 2**; **Figure S7**). The results suggest that the native range in North America has remained largely unchanged. In contrast, the introduced range in Europe is projected to experience a slight contraction in suitable habitats under future climatic conditions. In the eastern coastal regions of Oceania, where the species is currently absent, there is a slight expansion of suitable habitats. In East Asia, where the species has already invaded, there is no significant change in suitable habitats projected under future climatic conditions.

**Figure 3:**
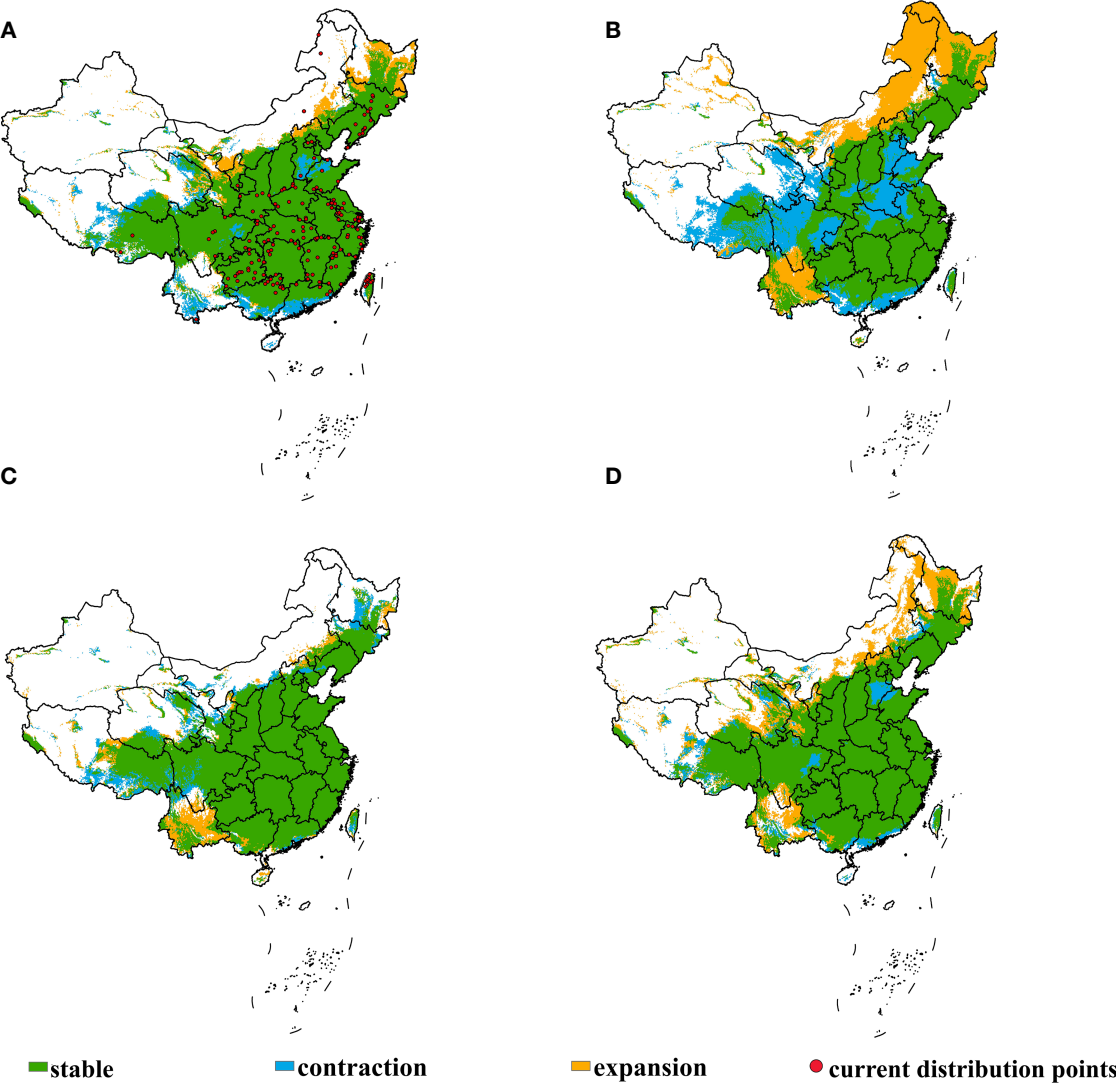
Shifts in potential suitable habitats of *Erigeron annuus* in China under different climate scenarios. **(A)** Current to SSP126, **(B)** current to SSP245, **(C)** current to SSP370, and **(D)** current to SSP585.

The study depicted the shifts of the potential suitable habitats of *E. annuus* in China under historical condition and different future climate scenarios in 2050, which are shown in **Figure 3**; **Table 2**; it was found that within China, most of the central, eastern, and southern regions were suitable for its growth (**Figures S4, S5**; **Table S1**). Specifically, under the SSP245 climate scenario, the suitable habitats of *E. annuus* exhibited notable expansion and contraction compared to historical conditions. The regions experiencing expansion primarily included Heilongjiang, Inner Mongolia, and Yunnan, and contraction areas were Tibet, Sichuan, Qinghai, Gansu, Henan, Hebei, and Shandong.

**Table 2:** Dynamics of the potential suitable habitats area of *Erigeron annuus* and *Erigeron philadelphicus* under different climate scenarios in China (compared to results with historical climate conditions).

Species	Climate scenario	Area (×10^4^ km^2^)			
		Expansion	Contraction	Stable	Change
*Erigeron annuus*	2050-SSP126	54.10	48.17	404.04	5.93
	2050-SSP245	155.80	150.08	302.14	5.72
	2050-SSP370	41.65	47.06	405.15	5.41
	2050-SSP585	101.83	36.85	415.36	64.98
*Erigeron philadelphicus*	2050-SSP126	34.82	17.24	307.81	17.58
	2050-SSP245	31.49	19.01	306.04	12.48
	2050-SSP370	31.01	17.03	308.01	13.98
	2050-SSP585	58.54	7.82	317.23	50.72

**Figure 4:**
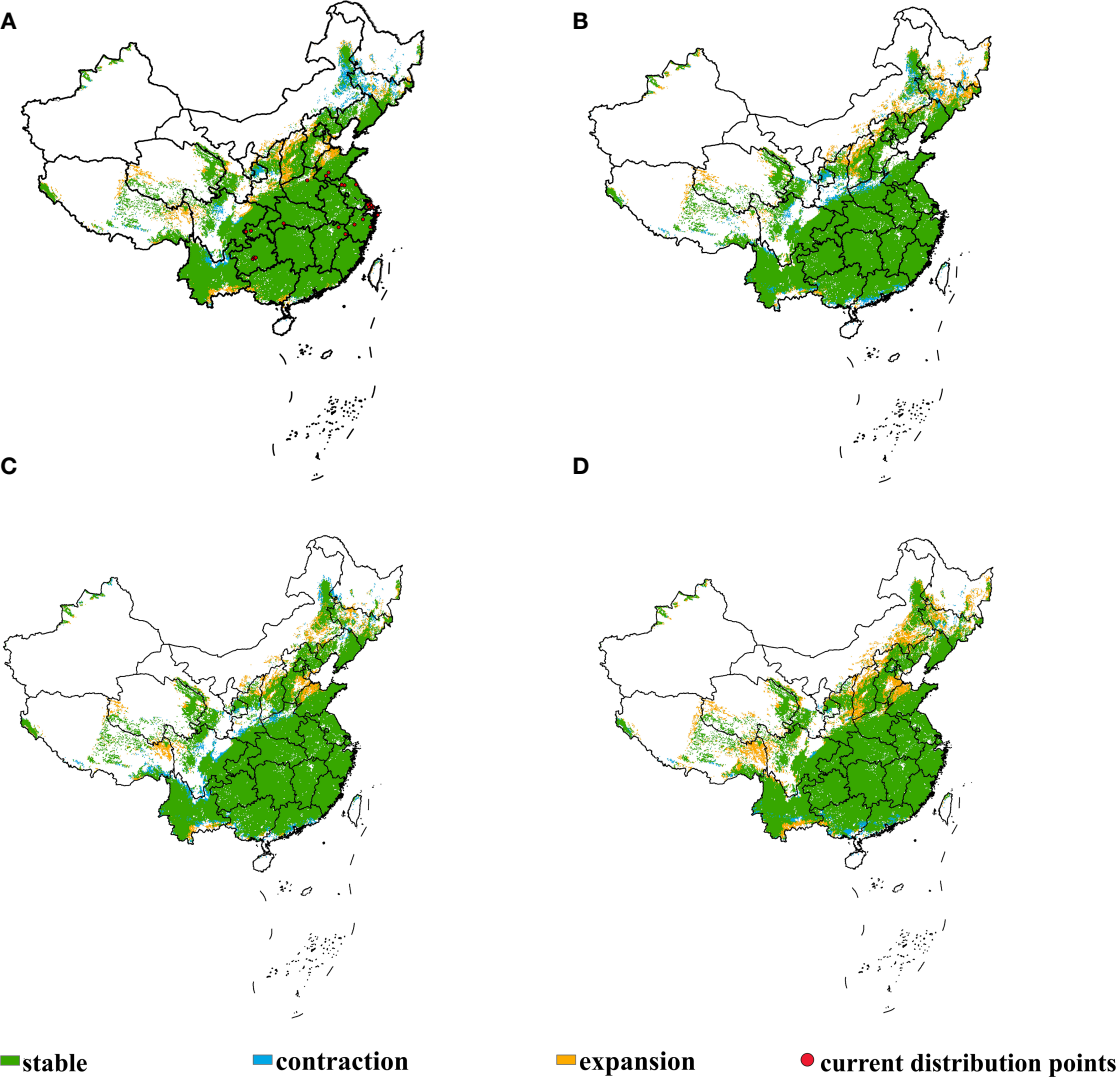
Shifts in potential suitable habitats of *Erigeron philadelphicus* in China under different climate scenarios. **(A)** Current to SSP126, **(B)** current to SSP245, **(C)** current to SSP370, and **(D)** current to SSP585.

The shifts of the potential suitable habitats of *E. philadelphicus* in China under historical climate condition and different future climate scenarios in 2050 are shown in **Figure 4**; **Table 2**, and it was found that within China, the range of suitable habitats was similar to the *E. annuus* but slightly smaller (**Figures S8, S9**). The overall potential suitable areas for *E. philadelphicus* are projected to expand mainly in regions such as Tibet, Shanxi, Shandong, Hebei, and Inner Mongolia, while the contraction area was less obvious compared with the historical climate scenario under climate change.

### Model validation

For the prediction of potential suitable habitats of *E. annuus*, five environmental variables were screened for *E. annuus*, the temperature seasonality (bio4), the precipitation of the wettest quarter (bio16), the precipitation of the driest month (bio14), the mean temperature of the wettest quarter (bio8), and the precipitation seasonality (bio15), contributing 29.9%, 27.4%, 23.4%, 16.8%, and 2.4%, respectively (**Table 3**). Analysis using the Jackknife method indicated that when considering “With only variable”, the precipitation of the driest month (bio14) had the highest gain, indicating that this variable contained the most effective information and had the most predictive ability; in the “Without variable” condition, ignoring the temperature seasonality (bio4) minimized the gain, indicating that this variable contained the most information that was not present in the other variables (**Figure S1A**).

**Table 3:** Contribution rate of selected environmental variables to the prediction of the potential suitable habitats of *Erigeron annuus* and *Erigeron philadelphicus*.

Species	Variable	Percent contribution	Permutation importance
	bio4	29.9	33.5
	bio16	27.4	19.7
*Erigeron annus*	bio14	23.4	3.7
	bio8	16.8	40
	bio15	2.4	3.1
	bio14	46.6	25.7
	bio7	27	37
*Erigeron philadelphicus*	bio13	19	22.2
	ESP	3.8	3.9
	ECE	3.5	11.3

The model prediction for *E. philadelphicus* included five selected variables: the precipitation of the driest month (bio14), the temperature annual range (bio7), the precipitation of the wettest month (bio13), the topsoil sodicity (ESP), and the topsoil salinity (ECE) contributing 46.6%, 27%, 19%, 3.8%, and 3.5%, respectively (**Table 3**). Jackknife analysis revealed that under the condition “With only variable”, the precipitation of the driest month (bio14) had the highest gain. Excluding the temperature annual range (bio7) resulted in minimal gain when analyzing the gain in the “Without variable” condition (**Figure S1B**).

**Figure 5:**
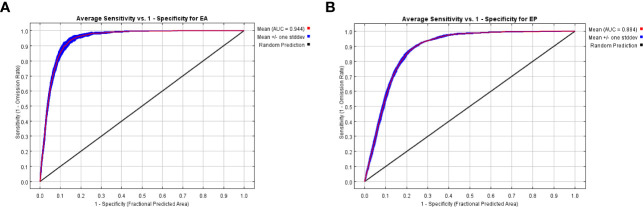
ROC curves and AUC values for predicted results. **(A)** *Erigeron annuus.* **(B)** *Erigeron philadelphicus*.

After optimization of FC and RM parameters, as shown in **Figure 5**, for *E. annus*, the AUC value was 0.944, indicating an excellent level of model performance; for *E. philadelphicus*, the AUC value was 0.8844, suggesting a good model performance.

### The shifts of distribution centroid and distribution pattern

In this study, we aimed to predict the shifts in distribution centroids of the two invasive alien weeds and compare the overlap and different areas of potential suitable habitats of *E. annuus* and *E. philadelphicus* to reveal the invasion trend of the two species within China.

**Figure 6:**
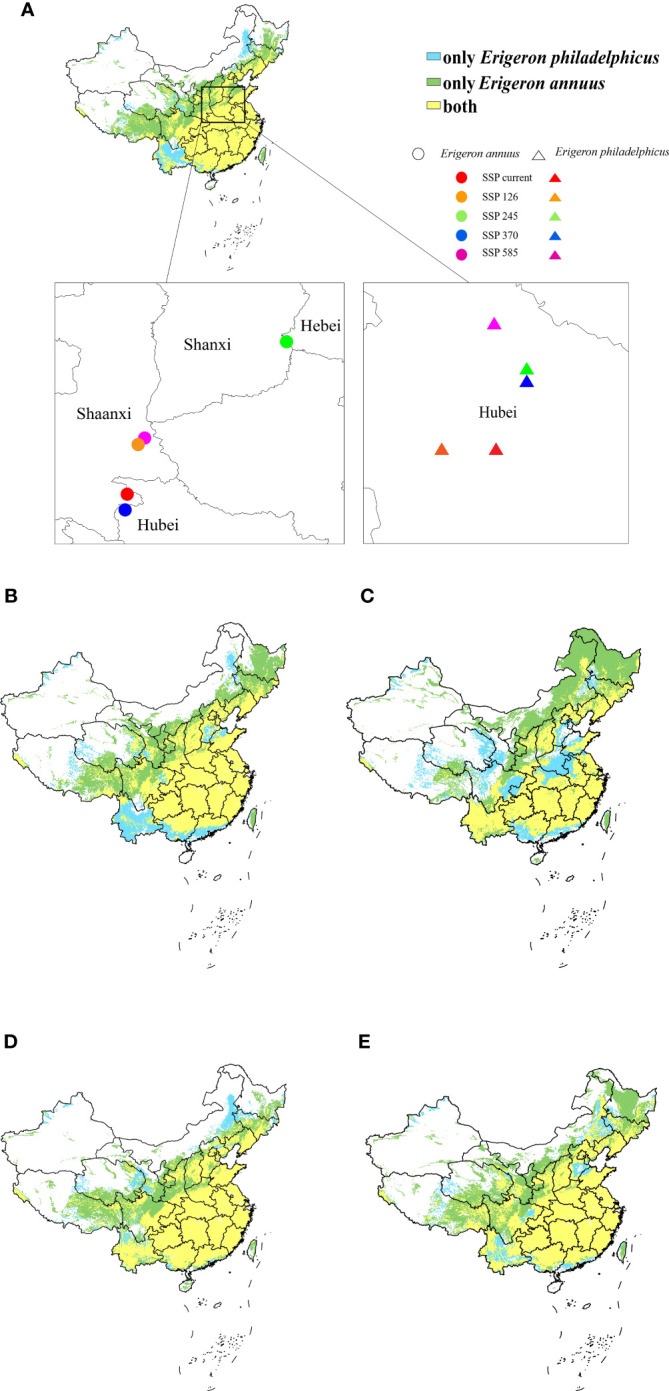
The shifts of distribution centroids and distribution pattern of two invasive *Erigeron* weeds under different climate scenarios. **(A)** History scenario, **(B)** 2050-SSP126, **(C)** 2050-SSP245, **(D)** 2050-SSP370, and **(E)** 2050-SSP585.

**Figure 6A** illustrates the trend of centroid transfer of *E. annuus* and *E. philadelphicus* in China from the current climate to the four scenarios (SSP126, SSP245, SSP370, and SSP585) in 2050. The distribution centroids for *E. annuus* showed a northward spreading trend shifting from Hubei to Hebei Province under SSP245 while the distribution centroids of *E. philadelphicus* were concentrated in Hubei Province. There was a sizable overlap between suitable habitats of *E. annuus* and *E. philadelphicus* in southern China. The potential suitable habitats for only *E. annuus* was larger than that of *E. philadelphicus* and mainly in the northern end of the overlapping areas (**Figure 6**). Additionally, the potential suitable habitats for only *E. philadelphicus* was mainly in Yunnan Province under historical climate condition, but overlapped with suitable habitats for *E. annuus* under future climate scenarios (**Figure 6**).

## Discussion

### Potential suitable habitats and significant variables

We identified topsoil salinity (Ece) and topsoil sodicity (Esp) as key factors influencing suitable areas for *E. philadelphicus*, which was consistent with observations that this species lacks resistance to salt and sodium. The results indicate that *E. philadelphicus* exhibits a preference for humid conditions, and according to the results, the precipitation of the driest month (bio14) was the variable with the highest contribution rate for this species. Conversely, *E. annuus* demonstrated higher adaptability and the ability to survive in poor soils, even growing in stone walls. Compared with previous studies, this study added soil variables into consideration, which greatly affect the distribution of weeds, but the collected distribution points may not cover all habitats where it is distributed, so there was still limitation in the prediction results.

We found that climate change has a similar effect on the distribution of *E. annuus* in its native range of North America and its introduced range in Europe and Asia. Overall, there is a noticeable expansion trend, but the general area of suitable habitat remains unchanged. On the other hand, for *E. philadelphicus*, we observed that there was no significant change in its distribution, both in its native range and in its introduced range. These results support the hypothesis of ecological niche conservatism in the case of *E. philadelphicus*. According to the ecological niche conservatism hypothesis, species tend to maintain their original ecological niche and adaptability as much as possible when facing climate change, resulting in a relatively slow response to environmental changes (Bates and Bertelsmeier, 2021). Therefore, when a species exhibits a higher degree of ecological niche conservatism, its range may remain relatively stable under climate change without significant changes. However, these results may also be influenced by other factors such as habitat suitability, competition, and human disturbance. Further research is needed to explore the mechanisms of species distribution changes and the role of ecological niche conservatism in the context of climate change.

The prediction results indicate that both weeds have extensive suitable habitats in China. *E. annuus*, being more adaptable, is expected to have a wider range of highly suitable areas including Fujian, Guizhou, and Taiwan. The southeast area will be the most suitable area for both weeds, indicating high invasive risk; *E. annuus* even shows a tendency to spread in the northeast area. Thus far, the current distributions of these two weeds were far from saturated in their suitable habitats, and appropriate quarantine control measures should be taken. Since both weeds are highly prolific in terms of seed production and rely on wind-borne spread, it is advisable to eradicate them before flowering (Zhang, 2011a; Pacanoski, 2017).

Both of these species originated from North America, and it is worth noting that North America and China have similar climatic zones south of 40° N. Thus, most North American species introduced to China adapt easily to their new habitat and establish themselves effectively in a relatively short period of time (Peel et al., 2007). Given this, it is imperative to enhance the prevention and control of quarantine at ports to prevent further introduction of these two invasive alien weeds.

The MaxEnt models were optimized using the R package “Enmeval”, resulting in an AUC value of 0.944 for *E. annuus* and 0.884 for *E. philadelphicus*. The predicted range of suitable habitats by the MaxEnt model aligned well with its historical distribution range, indicating that the models showed a good predictive ability. In this study, modeling primarily relied on known distribution records and climate data, but due to the diversity and complexity of climate change, the distribution data and climatic variables may still have some limitations. Additional factors, such as slope, slope orientation, soil capacitance, and organic matter content (Zhang et al., 2022a), were not taken into consideration. Furthermore, the invasion patterns of exotic organisms will be affected not only by climate but also by natural enemies, interspecific interactions, human interference, and other factors; thus, in the future, model prediction should encompass a broader range of factors to optimize the model evaluation and enhance the accuracy of prediction results (Jia et al., 2020).

### The distribution pattern and the shifts of distribution centroid

When comparing the shifts in potential suitable habitats of two invasive alien weeds under different climate scenarios separately, it was observed that both species are expected to expand globally. The SSP.2-4.5 climate scenario, with 3°C of warming, is projected to result in the largest expansion of *E. annuus.* This may be due to the increasing temperature favoring its physiology. However, if temperatures rise even higher, they may inhibit its growth, but the specific mechanism remains to be studied. The expansion of *E. philadelphicus* showed minimal change under four climate scenarios. Within China, the potential suitable areas of both invasive alien *Erigeron* weeds expanded the most under the SSP585 climate scenario. These results indicate that the change of suitable habitats in response to climate change is not a simple linear relationship. Therefore, it is crucial to strengthen the monitoring and prevention in provinces with suitable habitats.

The current climate and future scenarios indicate that the distribution centroid of *E. philadelphicus* is predicted to primarily reside in Hubei and is unlikely to move much under the climate change scenarios we examined. In contrast, the distribution centroid of *E. annuus* transitioned to the north from Hubei to Hebei under SSP245 scenarios, indicating higher adaptability to climate change. Thus, careful monitoring of potential range expansion of *E. annuus* in China is warranted.

By comparing the ecological niche overlap of the two weeds, it was found that the potential suitable habitats of *E. annuus* is projected to have a wider global distribution of potential suitable habitats, primarily in North America and Asia. Within China, it is predicted that *E. annuus* may extend to the northeastern region, and will invade parts of Yunnan Province with climate change that were suitable only for *E. philadelphicus* under historical climate conditions. Hence, it can be speculated that the invasive potential of *E. annuus* is relatively stronger. However, it remains to be studied whether the two weeds interfere with or complement each other during the invasion process. Both weeds have been established in China and caused losses, due to the diverse geography, climatic conditions, and farmland in China, and therefore reasonable and effective methods should be taken for their prevention and control to minimize losses.

## Conclusion

In this study, we optimized the MaxEnt model to predict the potential suitable habitats and distribution pattern of two invasive alien weeds *E. annuus* and *E. philadelphicus* under climate change. The predictions were based on the occurrence and related environmental variables. In addition to climate variables, potential suitable areas for *E. philadelphicus* were influenced by topsoil sodicity and topsoil salinity. The suitable habitats of the two invasive alien *Erigeron* weeds will expand under climate change. *E. annuus* exhibited the greatest expansion in its potential geographical distribution under the climate scenario SSP.2-4.5, which corresponds to a projected warming of 3.0°C in the future. Under future climate conditions, the centroid of *E. annuus* is projected to shift towards higher latitudes, whereas *E. philadelphicus* is expected to remain concentrated in Hubei Province. The overlap in suitable areas for both invasive alien *Erigeron* weeds is primarily observed in Jiangsu, Zhejiang, Fujian, Jiangxi, Hunan, Guizhou, and Chongqing, within China. Unlike *E. philadelphicus*, *E. annuus* may extend its range in areas such as Yunnan Province due to the climate change. Our research results provide support for careful monitoring and management of these two weed species in China under the influence of climate change.

## Data availability statement

The raw data supporting the conclusions of this article will be made available by the authors, without undue reservation.

## Author contributions

YH and YQ conceived and designed the research. YH, YZ, ZZ, WF, and YQ analyzed the data and wrote the first draft. GZ, ZL, and YQ discussed the idea and reviewed the draft. All authors revised the manuscript and approved the final version.
