# Supplementary material for: Impacts of climate change on climatically suitable regions of two invasive Erigeron weeds in China
Source: Front Plant Sci. 2023 Sep 28;14:1238656. doi: 10.3389/fpls.2023.1238656 (PMC10569594; doi:10.3389/fpls.2023.1238656)
Supplement: Supplementary file 1 [file DataSheet_1.docx]

Supplementary Material

**Assessing the climate change impacts on the habitat suitability and distribution pattern of two invasive *Erigeron* weeds**

**Yumeng Huang, Guoliang Zhang, Weidong Fu, Yue Zhang, Zihua Zhao, Zhihong Li, Yujia Qin**

* Corresponding author: E-mail: [qinyujia@cau.edu.cn](mailto:qinyujia@cau.edu.cn)

**
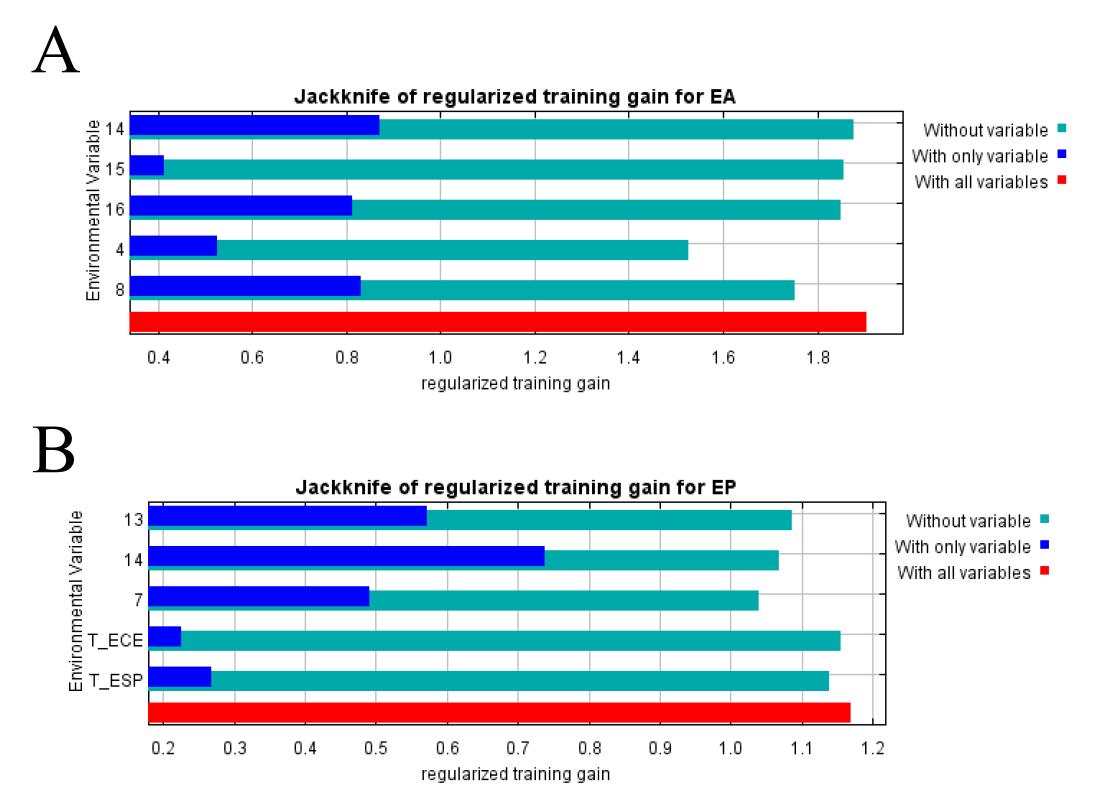
**

**Supplementary Figure 1.** Jackknife analysis of two invasive *Erigeron* weeds

Note:(A) *Erigeron annuus*; (B) *Erigeron philadelphicus*


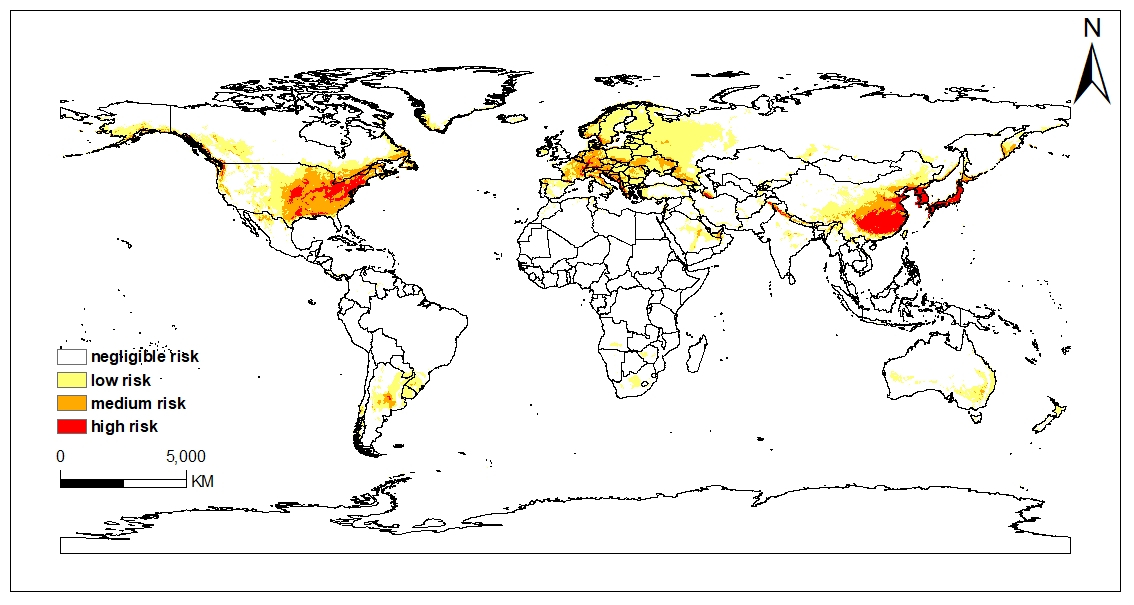


**Supplementary Figure 2.** Potential global suitable habitats of *Erigeron annuus* under historical climate condition


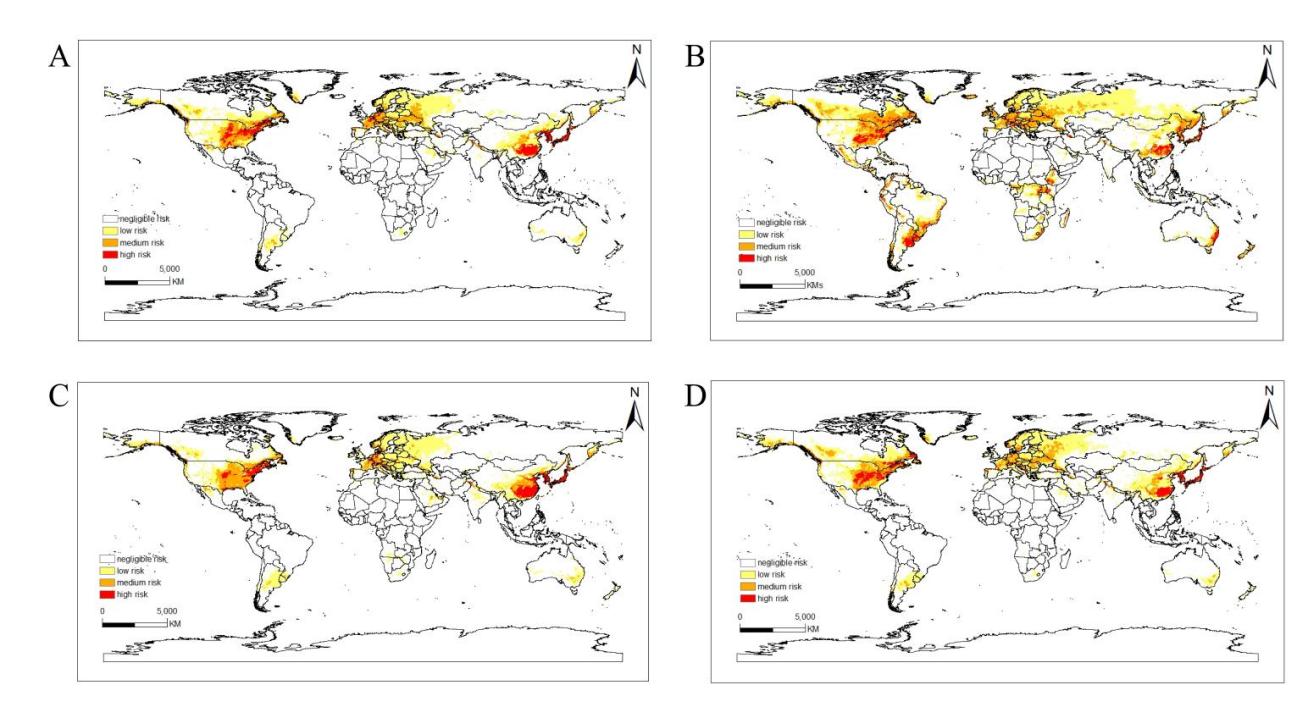


**Supplementary Figure 3.** Potential global suitable habitats of *Erigeron annuus* in 2050 under four future climate scenarios

Note:(A) SSP126, (B) SSP245, (C) SSP370, (D) SSP585


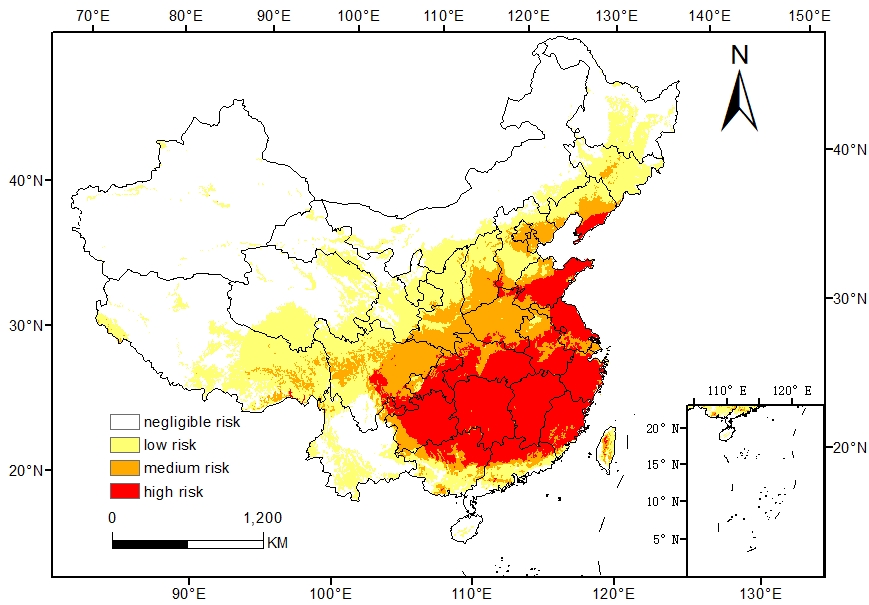


**Supplementary Figure 4.** Potential geographic distribution of *Erigeron annuus* in China under historical climate condition


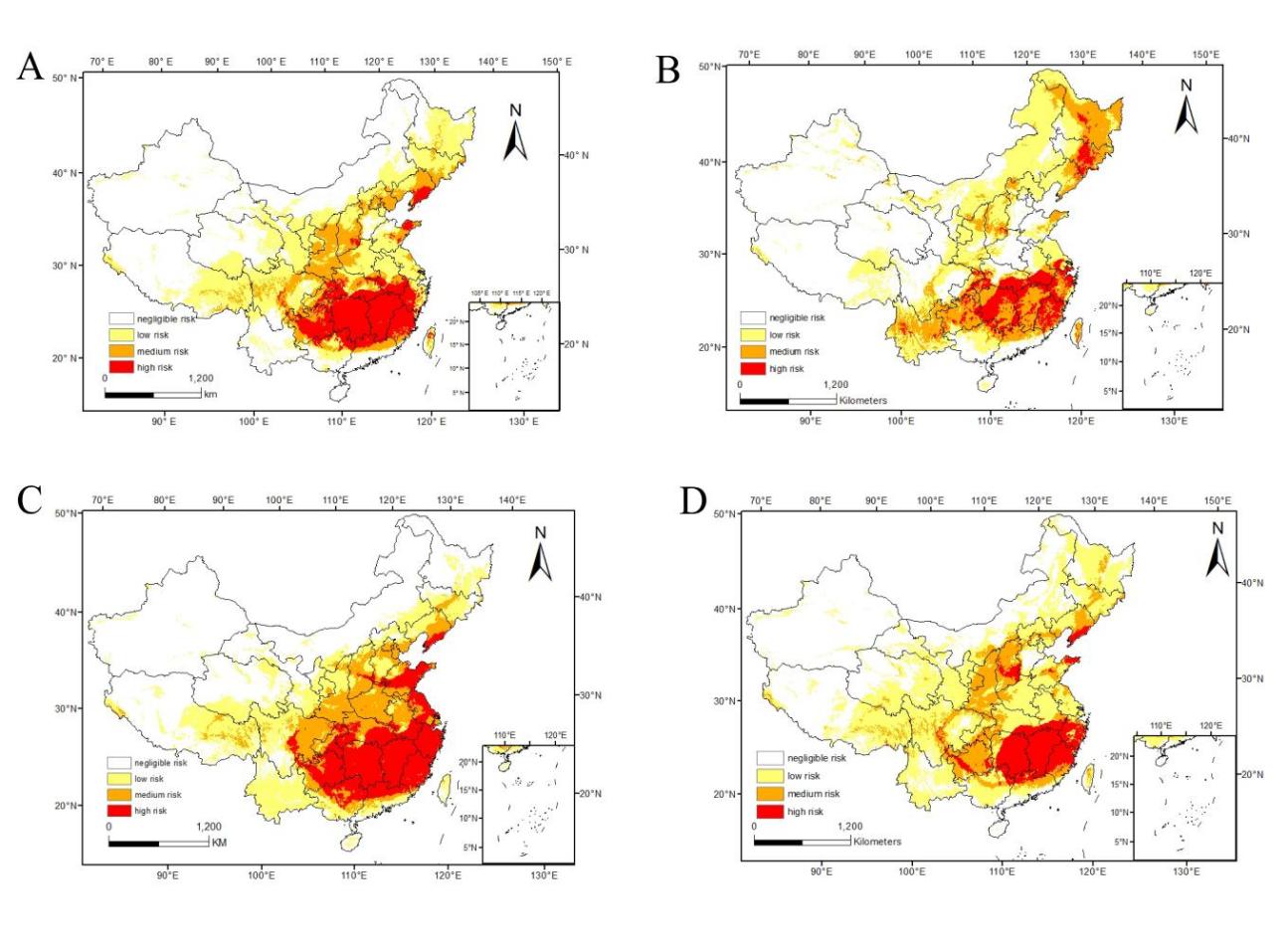


**Supplementary Figure 5.** Potential suitable habitats of *Erigeron annuus* in China in 2050 under four future climate scenarios

Note:(A) SSP126, (B) SSP245, (C) SSP370, (D) SSP585


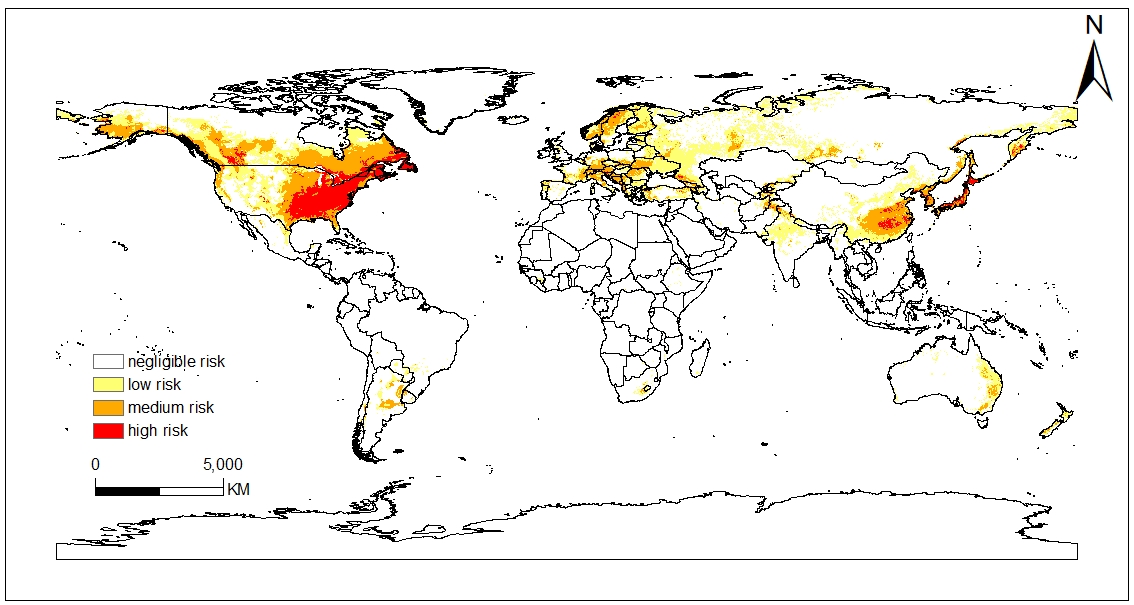


**Supplementary Figure 6.** Potential global suitable habitats of *Erigeron philadelphicus* under historical climate condition


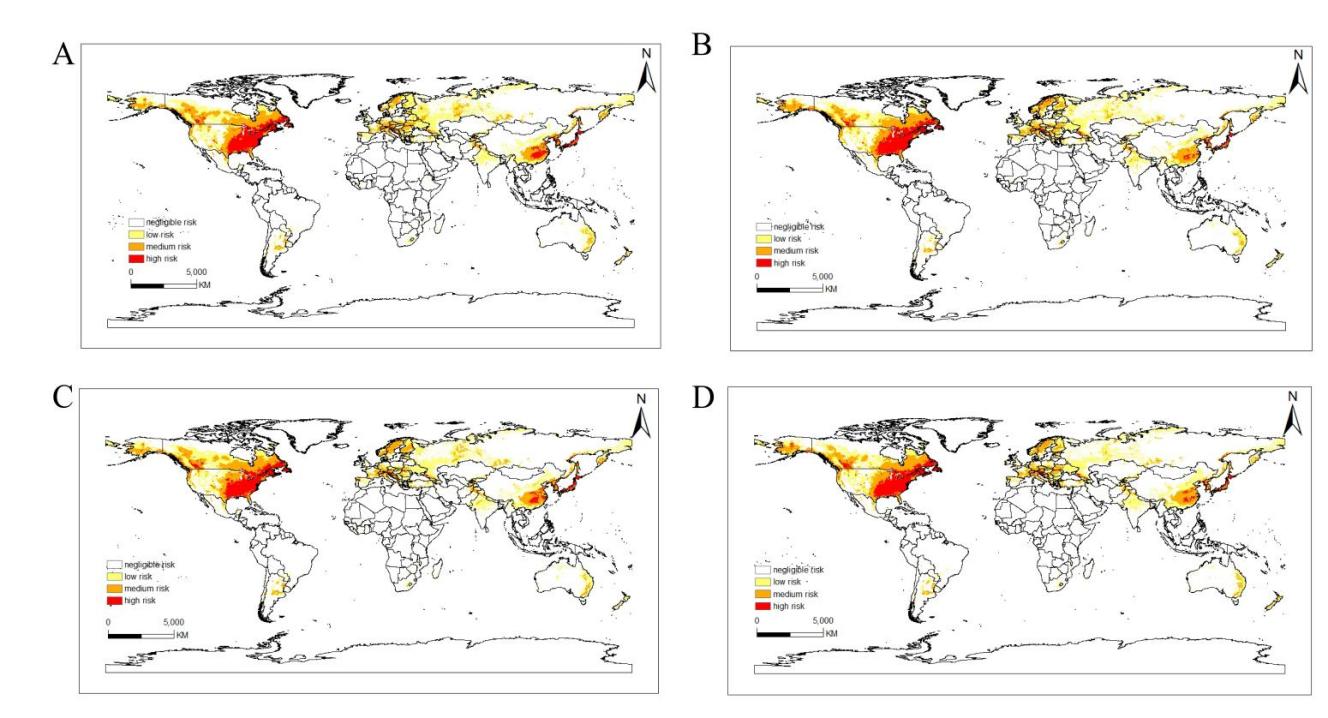


**Supplementary Figure 7.** Potential global suitable habitats of *Erigeron philadelphicus* in 2050 under four future climate scenarios

Note:(A) SSP126, (B) SSP245, (C) SSP370, (D) SSP585


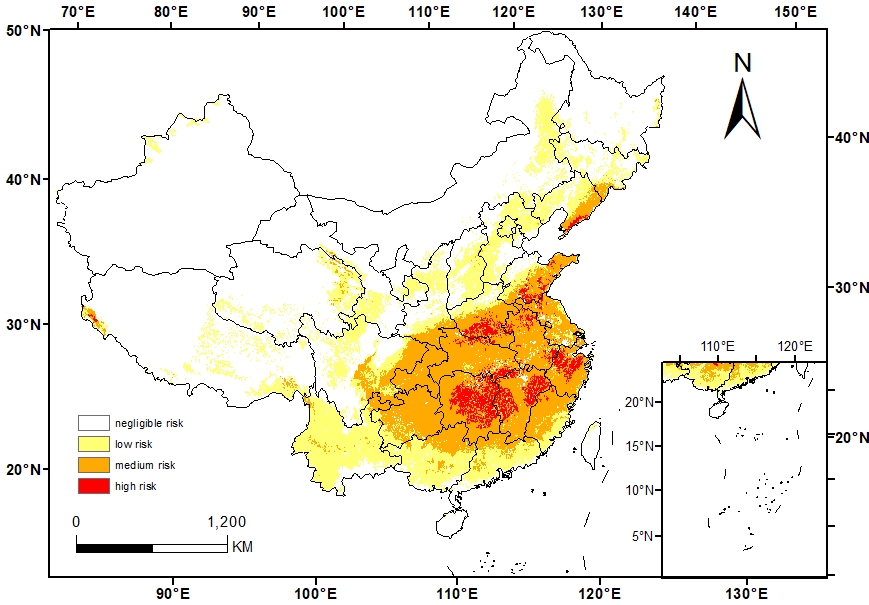


**Supplementary Figure 8.** Potential geographic distribution of *Erigeron philadelphicus* in China under historical climate

Condition


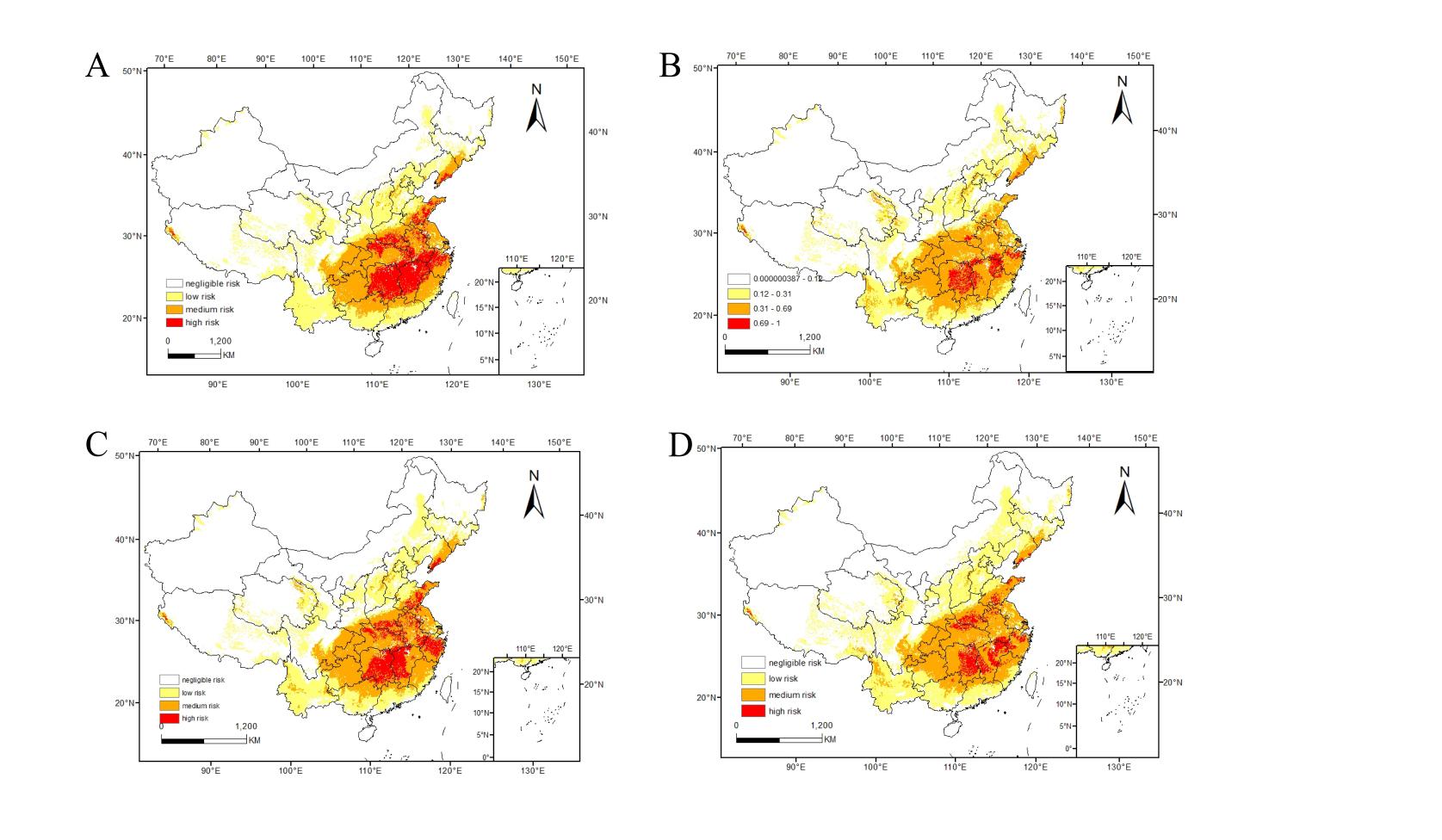


**Supplementary Figure 9.** Potential suitable habitats of *Erigeron philadelphicus* in China in 2050 under four future climate scenarios

Note:(A) SSP126, (B) SSP245, (C) SSP370, (D) SSP585

**Supplementary Table 1.** Suitable habitats area of *Erigeron annuus* and *Erigeron philadelphicus* in China under different climatic scenarios (in ten thousand km^2^)

| Species | Climate scenarios | Low risk | Medium risk | High risk |
| --- | --- | --- | --- | --- |
|  | Historical condition | 217.99 | 102.13 | 132.94 |
| *Erigeron annus* | 2050-SSP126 | 265.95 | 103.33 | 89.63 |
|  | 2050-SSP245 | 263.60 | 136.76 | 53.94 |
|  | 2050-SSP370 | 209.03 | 116.42 | 122.22 |
|  | 2050-SSP585 | 339.61 | 109.84 | 68.42 |
|  | Historical condition | 167.26 | 136.590 | 21.90 |
| *Erigeron philadelphicus* | 2050-SSP126 | 171.11 | 122.74 | 49.58 |
|  | 2050-SSP245 | 177.05 | 145.40 | 15.92 |
|  | 2050-SSP370 | 169.37 | 131.99 | 38.53 |
|  | 2050-SSP585 | 210.82 | 142.22 | 23.45 |
